# The Association between Whole Grain Products Consumption and Successful Aging: A Combined Analysis of MEDIS and ATTICA Epidemiological Studies

**DOI:** 10.3390/nu11061221

**Published:** 2019-05-29

**Authors:** Alexandra Foscolou, Nathan M. D’Cunha, Nenad Naumovski, Stefanos Tyrovolas, Christina Chrysohoou, Loukianos Rallidis, Antonia-Leda Matalas, Labros S. Sidossis, Demosthenes Panagiotakos

**Affiliations:** 1 Department of Nutrition and Dietetics, School of Health Science and Education, Harokopio University, 176 76 Athens, Greece; alexandra.foscolou@gmail.com (A.F.); s.tyrovolas@pssjd.org (S.T.); amatala@hua.gr (A.-L.M.); lss133@rci.rutgers.edu (L.S.S.); 2 Faculty of Health, University of Canberra, Canberra 2617, Australia; nathan.dcunha@canberra.edu.au (N.M.D.); Nenad.Naumovski@canberra.edu.au (N.N.); 3 Collaborative Research in Bioactives and Biomarkers (CRIBB) Group, University of Canberra, Bruce 2617, Australia; 4 Parc Sanitari Sant Joan de Déu, Fundació Sant Joan de Déu, CIBERSAM, Universitat de Barcelona, 08007 Barcelona, Spain; 5 First Cardiology Clinic, School of Medicine, University of Athens, 106 79 Athens, Greece; chrysohoou@usa.net; 6 Second Cardiology Clinic, School of Medicine, University of Athens, 106 79 Athens, Greece; lrallidis@gmail.com; 7 Department of Kinesiology and Health, School of Arts and Sciences, Rutgers University, New Brunswick, NJ 08901, USA

**Keywords:** whole grains, successful aging, fiber, older adults, Mediterranean, human health

## Abstract

The quality of carbohydrates in the diet, including whole grains, matters greatly to health. There is emerging evidence supporting various protective effects from whole grain consumption against certain chronic diseases. However, being free of disease is not a requirement for healthy ageing, as many older adults have one or more health conditions but, when well controlled, have little influence on their wellbeing. The present study aimed to evaluate the association between whole grain consumption on successful aging, through an analysis of a sample of *n* = 3349, over-50-years-old men and women participating in the ATTICA and MEDIS population-based cross-sectional studies. Successful aging was evaluated using the validated successful aging index (SAI, range 0–10) comprising of health-related social, lifestyle and clinical components. High whole grain intake was positively associated with SAI as compared with low (b ± SE: 0.278 ± 0.091, *p* = 0.002), whereas no significant associations were observed between moderate whole grain consumption and SAI (*p* > 0.05). Increased whole grain intake has been associated with several health benefits, and, as is shown here, with higher successful aging levels. Therefore, consumption of whole grains should be encouraged, especially by replacing refined grains, without increasing total energy intake.

## 1. Introduction

The consumption of whole grains has long been associated with reduced all-cause mortality, mainly by reducing the risk of overweight and obesity, type 2 diabetes, cardiovascular disease (CVD) and, possibly, colorectal cancer, in diverse populations [1,2,3]. The consumption of whole grains as part of a healthy dietary pattern has been widely supported by a number of nutrition organizations [4,5]. These beneficial effects are predominately ascribed to the composition of whole grains, which are reported to contain relatively high levels of potentially health-promoting compounds such as dietary fiber, resistant starch, and oligosaccharides. In addition, the majority of whole grains are also rich in several different antioxidants, including phenolic compounds and minerals as well as some other constituents such as phytates, phytoestrogens (lignan, plant stanols and sterols), B-vitamins and vitamin E [6,7]. Interestingly, the majority of these bioactives are removed during refining and cereal processing, with the final product containing significantly lower levels of these compounds [8]. Cereals, including oats, whole wheat, rye and millet are main sources of whole grains, which are associated with beneficial health outcomes across different age groups [9,10,11]. Due to their bioactive compounds and their effect on human health, high whole grain consumption is considered to promote disease prevention [1,2,6]. To date, several studies have attempted to investigate the relationship between whole grain consumption and various health outcomes [1,2,12,13]. Actually, according to a recent meta-analysis, consuming two to three servings of whole grains per day was associated with a 21% lower risk for cardiovascular events compared to a low daily consumption [14].

Successful aging is a multidimensional concept, referring to the avoidance of disease and disability and the maintenance of cognitive function, physical activity levels, and engagement in social and productive activities [15]. However, being free of disease is not a requirement for healthy aging, as many older adults have one or more health conditions that, when well controlled, have little influence on their wellbeing. Since successfully aging is quite a complex process, several physical activity and dietary practices have been recommended in order to achieve successful aging, including high consumption of whole grains [16]. Even though high consumption of whole grains is advantageous throughout the lifespan, it may be even more necessary for older people, due to the inverse association between whole grain consumption and inflammation, which is strongly involved in the onset of chronic diseases in that population [17].

There is a growing level of evidence supporting whole grain consumption as a major component of various healthy dietary patterns. For example, one of the most widely studied patterns, the Mediterranean diet, includes a high intake of whole grains and has long been associated with a variety of beneficial effects of several diseases such as CVD, dementia and cancer, and therefore could be associated with successful aging [17]. However, whether these effects are due to whole grain intake, other constituents of this dietary pattern or the combination of all foods included remains under investigation. Other studies have also focused on the relationship of whole grain intake on individual parameters related to successful aging. For example, a study in a Korean population found that high cereal whole grain consumption was positively related to cognitive function in older females [18].

However, studies investigating the relationship of whole grain intake, specifically on successful aging are lacking in the literature. In that context, the present work aimed to evaluate the association between whole grain consumption and successful aging, taking into account the overall dietary habits among middle-aged and older individuals participating in two large-scale epidemiological studies conducted in the Mediterranean region: the ATTICA and the MEDIS.

## 2. Materials and Methods

### 2.1. Sample

Two cross-sectional, population-based, large-scale epidemiologic studies (i.e., the ATTICA [19] and the MEDIS (MEDiterranean Islands Study) [20]), conducted in Greece, were combined for the purposes of the present work. In particular, the ATTICA study was a population-based, observational survey conducted in Athens greater area, Greece, during 2001–2002, with a follow-up during 2012. At baseline, all *n* = 3042 men and women participants (18+ years) were free of CVD and cancer, as assessed through a detailed clinical evaluation by the study’s physicians. From the original sample, a sub-group of all *n* = 1128 men and women, aged >50 years, were studied for the purposes of the present work. In addition, the MEDIS study was a population-based, observational survey that enrolled *n* = 3138 older people from 26 Mediterranean islands of five countries during 2005–2017; individuals who resided in assisted-living centers, had a clinical history of CVD or cancer or had left the island for a considerable period of time during their life (i.e., >5 years) were excluded from the sampling. For the purposes of the present work, a total of all *n* = 2221 men and women participants, living in 20 Greek islands, were studied. For both studies, a group of trained health scientists (cardiologists, general practitioners, dietitians and nurses) collected all information using standard, validated questionnaires and clinical procedures.

### 2.2. Bioethics

The ATTICA study was approved by the Bioethics Committee of Cardiology Department, University of Athens Medical School, and was carried out in accordance with the Declaration of Helsinki (1989) of the World Medical Association. The MEDIS study was approved by the Institutional Ethics Board of Harokopio University (16/19 December 2006) and followed the ethical recommendations of the World Medical Association (52nd WMA General Assembly, Edinburgh, Scotland, October 2000). In both studies, participants were informed of the study aims and procedures and provided written informed consent for study participation prior to enrollment.

### 2.3. Measurements

The socio-demographic characteristics studied here were age (years), gender (male/female) and place of residence (Athens area or Greek islands). Smoking status was evaluated following standard procedures for observational studies. Particularly, current smokers were defined as those who smoked at least one cigarette or any type of tobacco per day at the time of the interview. Former smokers were defined as those who previously smoked but had quit within the previous year. Current and former smokers were combined as ever smokers. The remaining participants were defined as nonsmokers.

### 2.4. Physical Activity Levels

Physical activity was evaluated in metabolic equivalent (MET) minutes per week, using the shortened, translated and validated in the Greek version of the self-reported International Physical Activity Questionnaire (IPAQ) [21]. Those who reported at least three MET minutes per week were classified as physically active. All others were defined as physically inactive.

### 2.5. Anthropometric and Clinical Characteristics

Weight and height were measured using standard procedures, and body mass index (BMI) (in kg/m^2^) was then calculated. Overweight was defined as BMI between 25.0 and 29.9 kg/m^2^, while obesity was defined as BMI > 29.9 kg/m^2^. Type 2 diabetes mellitus (T2DM) was determined by measuring fasting plasma glucose in accordance with the American Diabetes Association diagnostic criteria (fasting blood glucose > 126 mg/dL or use of antidiabetic medication). Participants who had blood pressure levels > 140/90 mmHg or who were administered antihypertensive medications were classified as hypertensive. Fasting blood lipids levels were also recorded. Hypercholesterolemia was defined as total serum cholesterol levels > 200 mg/dL, or the use of lipid-lowering agents, according to the National Cholesterol Education Program Adult Treatment Panel III guidelines [22]. The coefficient of variation for the blood measurements was <5%. Finally, a cumulative score of overall cardiometabolic risk, indicating the overall burden of four clinical CVD risk factors (i.e., obesity, hypertension, T2DM and hypercholesterolemia), was constructed (score range 0–4), wherein participants having none of the aforementioned risk factors were assigned a score of 0, having one factor a score of 1, etc.

### 2.6. Dietary Habits Assessment

In both studies, dietary habits were evaluated based on the participants’ responses on validated semi-quantitative food-frequency questionnaires (FFQ). Information regarding the frequency of all food groups and beverages, including whole or refined grain products (i.e., cereal, bread, pasta), as well as fast-food consumption was collected, based on “daily”, “weekly” (i.e., 1–2, 3–5 times per week), “monthly” basis (i.e., 2–3 times per month), “rare”, or “never”. Particularly, whole grain consumption was considered “low” when whole grain frequency intake was “rare” or “never”, “moderate” when whole grain frequency intake was two to three times per month or one to two times per week and, finally, “high” when whole grain consumption was four times per week or more. Specifically, among the ATTICA study participants, evaluation of dietary habits was based on a semi-quantitative FFQ, originally developed for the European Prospective Investigation into Cancer and Nutrition (EPIC) study [23]. The Greek version of the EPIC questionnaire was provided by the Unit of Nutrition of University of Athens Medical School, after being translated according to standard literature guidelines. Similar to the ATTICA study, dietary habits in the MEDIS study were assessed through a semi-quantitative, validated and reproducible FFQ developed for the study [24]. In both studies, participants were requested to report the average intake (per week or per day) of several food items and beverages that they had been consuming (during the last 12 months). The MedDietScore (range 0–55) evaluated the level of adherence to the Mediterranean diet [25].

### 2.7. Successful Aging Index

A successful aging index (SAI) was applied to evaluate the level of successful ageing of the participants ranging from 0 to 10, which has been previously developed and validated [20] using 10 attributes that reflected and were found to be associated with the aging process. In particular, SAI encompasses health-related social, lifestyle and clinical factors, including education, financial status, physical activity, BMI, depression, participation in social activities with friends and family, number of yearly excursions, total number of clinical CVD risk factors (i.e., history of hypertension, diabetes, hypercholesterolemia, obesity) and level of adherence to the Mediterranean diet.

### 2.8. Statistical Analysis

Continuous variables are presented as mean ± standard deviation (SD), and categorical variables as frequencies. Associations between continuous variables and group of whole grain consumption (i.e., low, moderate, high) were evaluated with the analyses of variance (ANOVA). Spearman’s Rho correlation coefficient was used to evaluate the relationships between whole grain consumption and SAI score. Linear regression analysis was further used to evaluate the association between whole grain consumption (evaluated as a categorical variable, “low” vs. “moderate”, and “low” vs. “high” consumption) (independent variables) and the SAI score (outcome), after adjusting for various participants’ characteristics (i.e., age, sex, smoking habits, physical activity level and carbohydrate intake as % energy). Linear regression analysis was also used to evaluate the association of whole grain intake consumption on CVD risk score (outcome), following adjustments for age, sex, smoking habits and level of adherence to the Mediterranean diet. Results are presented as unstandardized beta coefficients ± standard error and *p*-value. Linearity of the models’ fitting was tested through scatter plots of standardized residuals against fitted values; normality of regression residuals was evaluated through P-P plots; dependency was tested using a Durbin–Watson test and homoscedasticity using the variance inflation index (value < 4 suggests a lack of heteroscedasticity). STATA software version 13 (M. Psarros & Associates, Sparti, Greece) was used for all calculations.

## 3. Results

Various socio-demographic, lifestyle and clinical characteristics of the ATTICA and MEDIS study participants based on the whole grain consumption are presented in Table 1. Participants with high whole grain intake were more likely to be younger (*p* < 0.001), to consume more carbohydrates (*p* < 0.001), have a higher level of SAI (*p* < 0.001) compared with participants with moderate or low whole grain consumption, less likely to have hypertension compared to participants with moderate (*p* < 0.001) or low whole grain consumption (*p* = 0.013) and less likely to have diabetes compared with those with low whole grain consumption (*p* = 0.009). Participants with high whole grain intake were also more likely to be smokers and have hypercholesterolemia compared with participants with moderate (*p* < 0.001 and *p* = 0.007 for smoking and hypercholesterolemia, respectively) or low (*p* = 0.002 and *p* = 0.001 for smoking and hypercholesterolemia, respectively) whole grain consumption.

A significant correlation was observed between whole grain consumption (as servings per week) and SAI score (Spearman’s Rho = 0.227, *p* < 0.001); thus, to further evaluate the research hypothesis, multiple linear regression analysis was then applied. After adjustments (Table 2) for age, sex, smoking habits and carbohydrate consumption as percentage of total energy intake, “low” compared to “high” whole grain intake was inversely associated with SAI (b ± SE: −0.278 ± 0.091, *p* = 0.002), whereas no significant association was observed between “low” vs. “moderate” and “moderate” vs. “high” whole grain consumption and SAI (*p* = 0.901 and *p* = 0.062, respectively). The findings did not alter when the MedDietScore (a proxy of adherence to the Mediterranean diet) was included in the analyses (data not shown in tables).

Accordingly, a significant correlation was observed between whole grain consumption and CVD risk score (Spearman’s Rho = −0.101, *p* < 0.001). However, residual confounding may exist. Thus, multiple linear regression (Table 3) was used to evaluate the association between whole grain consumption and CVD risk factor score after adjustments for age, sex, physical activity, smoking habits and level of adherence to the Mediterranean diet. It was observed that “high” compared to “low” whole grain intake was inversely associated with CVD risk factor score (b ± SE: −0.125 ± 0.062, *p* = 0.044). No significant association was observed between “high” and “moderate” or “moderate” and “low” whole grain consumption and CVD risk (*p* = 0.352 and *p* = 0.402, respectively).

## 4. Discussion

The present study aimed to investigate the association between whole grain consumption on successful aging among older adults living in the Mediterranean region. Based on the observed results, higher intake of whole grains was associated with a higher level of successful aging, independently of participant’s age, sex or other dietary habits. In addition, high whole grain consumption was associated with lower CVD risk, irrespective of the influence of potentially confounding variables. The presented results deserve further attention from a public health point of view. Defining successful aging is not simple, and has long been the subject of vigorous inquiry and debate in medical science, and particularly in gerontology. Conceptually, one prominent model of successful aging, developed in the 1990s, included freedom from disease and disability, high cognitive and physical functioning and active engagement with life [15]. In recent years, gerontologists have gone beyond this model, but this construct is still relevant today. However, being free of disease is not a requirement for healthy ageing, as many older adults have one or more health conditions, but which have a minimal influence on their wellbeing when controlled through diet, lifestyle and appropriate medication.

Since no other studies have investigated the relationship of whole grain intake on successful aging (as a multivariable concept), head-to-head comparisons cannot be made. However, the beneficial effects of whole grain products consumption on human health are already established. The benefits are mainly attributed to the consumption of the whole grain (i.e., entire grain, bran and germ) [26], the amount of cereal fibers [27] and the higher proportion of several micronutrients and vitamins that whole grain products contain (i.e., phosphorus, thiamine, magnesium, niacin, vitamins E, B6, K, A, zinc, iron, potassium, riboflavin and calcium) compared to refined flour and grain products [6,28]. Additionally, the micronutrient composition of the whole grains (including their products), particularly B-vitamins such as folate, are valuable components in the prevention of dementia and Alzheimer’s disease [29], and consequently cognitive function—a basic component of successful aging. Nonetheless, it should be noted that the findings of this study concerning the association of whole grain intake on successful aging are in line with those of previous studies investigating the association of whole grain intake on some of the parameters related to successful aging [26,29]. It is also suggested that an increase in fiber intake could potentially lead to better aging trajectories, since whole grains and fiber help maintain normal body weight and achieve better lipid profile, endothelial function, glycemic control, insulin sensitivity and maintenance of digestive health [26,30,31].

Although overall grain consumption is increasing worldwide [32], whole grain intake is decreasing mainly due to the higher availability of ultra-processed grain food products, increasing trends related to the consumption of gluten-free foods, and the emergence and marketing of fad diets, particularly in developed countries [33,34]. Cereal grains have been one of the main components of the human diet for several thousands of years and have helped shape human civilization. In particular, wheat, maize and rice are considered to be the three cereal grains that contribute most to overall energy intake [32]. However, changes in taste, trends, costs and availability may also have an impact on dietary choices and behavior, consequently influencing the consumption of food and grains. Many cereal-producing countries such as China have turned towards consuming nontraditional diets consisting of a higher intake of meat and dairy products as well as fast food and packaged foods not cooked at home [35]. Overall, nutritious and healthy dietary patterns based more on higher consumption of fruits, nuts, vegetables and whole grain products (i.e., nutrient-rich plant foods), and less on foods high in saturated or trans fatty acids, and foods with added sugar and sodium, may also be climate-friendly [36]. For that reason, the promotion of traditional diets such as the Mediterranean diet containing whole grains is encouraged [37], since older people are a population at risk of disease onset both due to their age and due to their poor adherence to healthy dietary patterns.

Despite the importance of high whole grain consumption to promote successful aging, a major difficulty to be overcome concerns the limited financial resources of many older people, which debar the adoption of a healthy diet [38,39]. Educating older people on the benefits of consuming whole grain products might be insufficient to increase consumption. However, it should also be taken into account that older adults may have lower incomes than the population as a whole. Older individuals may be forced to cut back on several basic necessities, including the supply of food. It has been reported that foods high in salt, sugar, fat and generally lower in nutritional value are often the cheaper on the market due to low taxation and agricultural subsidies and that, therefore, older people may tend to consume these foods instead of healthier options [39]. However, by taxing unhealthy foods (i.e., foods high in saturated fats, excess salt, sugars) and subsidizing healthy foods (i.e., fruits, vegetables, whole grain products), it is possible to tip the pricing structure in favor of choosing healthier options. It can also provide an incentive to manufacturers of processed foods to improve the nutritional profiles of their products.

In general, the aforementioned propositions should be examined under the principle of the demographic transition taking place in most countries of the developed world. More specifically, rapid population aging trends are a major challenge for health systems, which have to deal with the increased cost of healthcare for the aging population. The continuous trend towards an aged population underlines the necessity to design interventions for older people. However, health-promoting interventions aiming to increase whole grain consumption should target all age groups, in order to increase the possibility that they will be functional and disease-free when reaching the third age, since morbidity developed during third age is strongly related to dietary behaviors through the lifespan. Overall, policy recommendations should be categorized into three actions, aiming, first of all, to increase the awareness of consumers regarding the potential benefits of whole grain products while also providing information on how to recognize the appropriate products; secondly, to make healthy options available by improving food environments (e.g., increasing the availability and palatability of whole grain products); and finally, to implement financial incentives to promote the purchasing of these healthful foodstuffs by consumers.

To the best of our knowledge, this is one of the first studies evaluating whole grain consumption in relation to successful aging in older people living in the Mediterranean region. Since two large-scale population-based studies were used, it is anticipated that the effects of population bias have been minimized and the external validity of findings is consequently augmented. However, this study has some limitations that should be taken into account before generalizing the results. The observational nature of the cross-sectional design does not allow for causal associations to be drawn. It is also not possible to discern between which foods have been displaced by the higher consumption of whole grains and, as such, the benefits may be partially attributed to lower consumption of nutrient-poor foods. Moreover, dietary habits were measured only once based on food frequency questionnaires and thus recall bias may exist. Successful aging is characterized by low probability of disease and related disabilities, high cognitive and physical functional capacity and active engagement with life; however, in the present study, successful aging was not evaluated through cognitive behavior, nor the mobility and functionality of the participants, which may constitute another limitation.

## 5. Conclusions

As supported by the results of the present study, higher whole grain intake was independently associated with higher successful aging levels and lower cardiometabolic risk for the MEDIS and the ATTICA study participants. The aforementioned findings are in line with previous evidence supporting a beneficial effect of whole grain intake on human health. For that reason, higher whole grain intake should be strongly promoted and encouraged as a part of healthy dietary patterns, especially by replacing refined grains without increasing total energy intake, in order to enhance the successful aging process.

## Figures and Tables

**Table 1:** Sociodemographic, lifestyle and clinical characteristics of the participants based on the weekly frequency of whole grain products.

		Whole Grains						
	All Participants	Low Intake	Moderate Intake	High Intake	*p*	*p* ^1^	*p* ^2^	*p* ^3^
*N*	3349	446	826	620				
Age (years)	69.2 ± 10.2	74.7 ± 7.4	74.9 ± 7.4	67 ± 10	<0.001	1.000	<0.001	<0.001
Male *n* (%)	1751 (52)	263 (59)	465 (56)	370 (60)	0.392	1.000	0.592	1.000
Ever smokers *n* (%yes)	1358 (43)	157 (41)	285 (38)	310 (52)	0.001	0.793	<0.001	0.002
Physically active *n* (%active)	1372 (41)	195 (44)	356 (44)	301 (49)	0.104	1.000	0.124	0.385
BMI (kg/m^2^)	28 ± 4	28 ± 4.2	28 ± 4.3	28 ± 4.0	0.645	1.000	1.000	1.000
Carbohydrate intake (g/day)	144 ± 68	118 ± 53	146 ± 53	193 ± 76	<0.001	<0.001	<0.001	<0.001
Whole grain intake (srv/week)	52 ± 45	21 ± 5.1	35 ± 5.7	96 ± 54	<0.001	<0.001	<0.001	<0.001
MedDietScore (0–55)	29 ± 7	29 ± 5.6	33 ± 4.8	31 ± 6.9	<0.001	<0.001	<0.001	<0.001
Hypertension *n* (%yes)	1881 (86)	271 (84)	528 (90)	311 (77)	0.001	0.093	<0.001	0.013
Diabetes *n* (%yes)	696 (21)	123 (28)	176 (22)	123 (20)	0.008	0.040	1.000	0.009
Hypercholesterolemia *n* (%yes)	1747 (53)	191 (43)	400 (49)	340 (55)	0.001	0.174	0.070	0.001
CVD risk factors (0–4)	1.6 ± 1.1	1.6 ± 1.1	1.7 ± 1.0	1.5 ± 1.1	0.134	1.000	0.137	1.000
SAI (0–10)	3.1 ± 1.2	2.9 ± 1.3	3.0 ± 1.3	3.4 ± 1.2	<0.001	1.000	<0.001	<0.001

Values are presented as percent (%) or mean ± standard deviation. *p*: *p*-value derived from analysis of variance (ANOVA) for continuous variables or the chi-square test for the categorical variables; *p*^1^: between “low intake” and “moderate intake” intake groups; *p*^2^: between “moderate intake” and “high intake” intake groups; *p*^3^ between “low intake” and “high intake” intake groups, after correcting for the inflation of type-I error with the Bonferroni rule. BMI = body mass index; SAI = successful aging index; srv = serving.

**Table 2:** Results from linear regression models that evaluated the association of whole grain intake on successful ageing (outcome) among ATTICA (n = 1128) and MEDIS (n = 2221) study participants.

	b ± SE	*p*
**Model for:** low vs. moderate whole grain intake	0.010 ± 0.083	0.901
**Model for:** low vs. high whole grain intake	−0.278 ± 0.091	0.002
**Model for:** moderate vs. high whole grain intake	−0.178 ± 0.095	0.062

Results are presented as b ± SE: unstandardized b coefficients ± standard error and *p*-value. All models were adjusted for age, sex, smoking habits and carbohydrate intake.

**Table 3:** Results from linear regression models that evaluated the association of whole grain intake on CVD risk factor score (outcome) among ATTICA (*n* = 1128) and MEDIS (*n* = 2221) study participants.

	b ± SE	*p*
**Model for:** high vs. low whole grain intake	−0.125 ± 0.062	0.044
**Model for:** high vs. moderate whole grain intake	−0.062 ± 0.067	0.352
**Model for:** moderate vs. low whole grain intake	0.053 ± 0.063	0.402

Results are presented as b ± SE: unstandardized b coefficients ± standard error and *p*-value. All models were adjusted for age, sex, physical activity status, smoking habits and adherence to the Mediterranean diet.

## References

[1] Aune D., Keum N., Giovannucci E., Fadnes L.T., Boffetta P., Greenwood D.C., Tonstad S., Vatten L.J., Riboli E., Norat T. (2016). Whole grain consumption and risk of cardiovascular disease, cancer, and all cause and cause specific mortality: Systematic review and dose-response meta-analysis of prospective studies. Br. Med. J..

[2] Sandberg J.C., Björck I.M.E., Nilsson A.C. (2018). Increased plasma brain-derived neurotrophic factor 10.5 h after intake of whole grain rye-based products in healthy subjects. Nutrients.

[3] Wu H., Flint A.J., Qi Q., van Dam R.M., Sampson L.A., Rimm E.B., Holmes M.D., Willett W.C., Hu F.B., Sun Q. (2015). Association between dietary whole grain intake and risk of mortality: Two large prospective studies in US men and women. JAMA Intern. Med..

[4] Mann J., Cummings J.H., Englyst H.N., Key T., Liu S., Riccardi G., Summerbell C., Uauy R., van Dam R.M., Venn B. (2007). FAO/WHO scientific update on carbohydrates in human nutrition: Conclusions. Eur. J. Clin. Nutr..

[5] U.S. Department of Health and Human Services and U.S. Department of Agriculture (2015). 2015–2020 Dietary Guidelines for Americans.

[6] Tovar J., Nilsson A., Johansson M., Björck I. (2014). Combining functional features of whole-grain barley and legumes for dietary reduction of cardiometabolic risk: A randomised cross-over intervention in mature women. Br. J. Nutr..

[7] Slavin J. (2004). Whole grains and human health. Nutr. Res. Rev..

[8] Slavin J.L., Jacobs D., Marquart L. (2001). Grain processing and nutrition. Crit. Rev. Biotechnol..

[9] Juan J., Liu G., Willett W.C., Hu F.B., Rexrode K.M., Sun Q. (2017). Whole grain consumption and risk of ischemic stroke: Results from 2 prospective cohort studies. Stroke.

[10] Kafatos A., Linardakis M., Bertsias G., Mammas I., Fletcher R., Bervanaki F. (2005). Consumption of ready-to-eat cereals in relation to health and diet indicators among school adolescents in Crete, Greece. Ann. Nutr. Metab..

[11] Sahyoun N.R., Jacques P.F., Zhang X.L., Juan W., McKeown N.M. (2006). Whole-grain intake is inversely associated with the metabolic syndrome and mortality in older adults. Am. J. Clin. Nutr..

[12] de Munter J.S., Hu F.B., Spiegelman D., Franz M., van Dam R.M. (2007). Whole grain, bran, and germ intake and risk of type 2 diabetes: A prospective cohort study and systematic review. PLoS Med..

[13] Huang T., Xu M., Lee A., Cho S., Qi L. (2015). Consumption of whole grains and cereal fiber and total and cause-specific mortality: Prospective analysis of 367,442 individuals. BMC Med..

[14] Mellen P.B., Walsh T.F., Herrington D.M. (2008). Whole grain intake and cardiovascular disease: A meta-analysis. Nutr. Metab. Cardiovasc. Dis..

[15] Rowe J.W., Kahn R.L. (1997). Successful aging. Gerontologist.

[16] Topp R., Fahlman M., Boardley D. (2004). Healthy aging: Health promotion and disease prevention. Nurs. Clin. N. Am..

[17] Jenny N.S. (2012). Inflammation in aging: Cause, effect, or both?. Discov. Med..

[18] Lee L., Kang S.A., Lee H.O., Lee B.H., Park J.S., Kim J.H., Jung I.K., Park Y.J., Lee J.E. (2001). Relationships between dietary intake and cognitive function level in Korean elderly people. Public Health.

[19] Panagiotakos D.B., Georgousopoulou E.N., Pitsavos C., Chrysohoou C., Metaxa V., Georgiopoulos G.A., Kalogeropoulos K., Tousoulis D., Stefanadis C., ATTICA Study Group (2015). Ten-year (2002–2012) cardiovascular disease incidence and all-cause mortality, in urban Greek population: The ATTICA Study. Int. J. Cardiol..

[20] Tyrovolas S., Haro J.M., Mariolis A., Piscopo A., Valacchi G., Tsakountakis N., Zeimbkeis A., Tyrovola D., Bountziouka V., Lionis C. (2014). Successful aging, dietary habits and health status of elderly individulas: A k-dimensional approach within the multi-national MEDIS study. Exp. Gerontol..

[21] Papathanasiou G., Georgoudis G., Papandreou M., Spyropoulos P., Georgakopoulos D., Kalfakakou V., Evangelou A. (2009). Reliability measures of the short International Physical Activity Questionnaire (IPAQ) in Greek young adults. Hell. J. Cardiol..

[22] Expert Panel on Detection, Evaluation, and Treatment of High Blood Cholesterol in Adults (2001). Executive summary of the third report of the National Cholesterol Education Program (NCEP) expert panel on detection, evaluation, and treatment of high blood cholesterol in adults (adult treatment panel III). J. Am. Med. Assoc..

[23] Katsouyanni K., Rimm E.B., Gnardellis C., Trichopoulos D., Polychronopoulos E., Trichopoulou A. (1997). Reproducibility and relative validity of an extensive semi-quantitative frequency questionnaire using dietary records and biochemical markers among Greek schoolteachers. Int. J. Epidemiol..

[24] Tyrovolas S., Pounis G., Bountziouka V., Polychronopoulos E., Panagiotakos D. (2010). Repeatability and validation of a short, semi-quantitative food frequency questionnaire designed for older adults living in Mediterranean areas: The MEDIS-FFQ. J. Nutr. Elder..

[25] Panagiotakos D.B., Pitsavos C., Stefanadis C. (2006). Dietary patterns: A Mediterranean diet score and its relation to clinical and biological markers of cardiovascular disease risk. Nutr. Metab. Cardiovasc. Dis..

[26] Calinoiu L.F., Vodnar D.C. (2018). Whole grains and phenolic acids: A review on bioactivity, functionality, health benefits and bioavailability. Nutrients.

[27] Curtain F., Grafenauer S. (2019). Health star rating in grain foods—Does it adequately differentiate refined and whole grain foods?. Nutrients.

[28] USDA United States Department of Agriculture. Agricultural Research Service. USDA Food Composition Databases. https://ndb.nal.usda.gov/ndb/.

[29] D’Cunha N.M., Georgousopoulou E.N., Boyd L., Veysey M., Sturm J., O’Brien B., Lucock M., McKune A.J., Mellor D.D., Roach P.D. (2019). Relationship between B-vitamin biomarkers and dietary intake with apolipoprotein E e4 in Alzheimer’s disease. J. Nutr. Gerontol. Geriatr..

[30] Gopinath B., Flood M.V., Kifley A., Louie C.Y.J., Mitchell P. (2016). Association between carbohydrate nutrition and successful aging over 10 years. J. Gerontol. A Biol. Sci. Med. Sci..

[31] Ma X., Tang W.G., Yang Y., Zhang Q.L., Zheng J.L., Xiang Y.B. (2016). Association between whole grain intake and all-cause mortality: A meta-analysis of cohort studies. Oncotarget.

[32] Jones J.M., Sheats D.B. (2016). Consumer trends in grain consumption. Reference Module in Food Science.

[33] Kim H., Hu E.A., Rebholz C.M. (2019). Ultra-processed food intake and mortality in the USA: Results from the Third National Health and Nutrition Examination Survey (NHANES III, 1988–1994). Public Health Nutr..

[34] Livesey G., Livesey H. (2019). Coronary heart disease and dietary carbohydrate, glycemic index, and glycemic load: Dose-response meta-analyses of prospective cohort studies. Mayo Clin. Proc. Innov. Qual. Outcomes.

[35] Nilsson A., Montiel Rojas D., Kadi F. (2018). Impact of meeting different guidelines for protein intake on muscle mass and physical function in physically active older women. Nutrients.

[36] Chen J., Fewtrell M., Kennedy G., Naska A., Riediger K., Roos N., Sanders T., Tuohy K.M., Valtuena-Martinez S. (2016). Nutrition challenges ahead. Special issue: EFSA’s 2^nd^ Scientific Conference Shaping the Future of Food Safety Together. EFSA J..

[37] Godos J., Castellano S., Marranzano M. (2019). Adherence to a Mediterranean dietary pattern is associated with higher quality of life in a cohort of Italian adults. Nutrients.

[38] Nooyens A.C., Visscher T.L., Schuit A.J., van Rossum C.T., Verschuren W.M., van Mechelen W., Seidell J.C. (2005). Effects of retirement on lifestyle in relation to changes in weight and waist circumference in Dutch men: A prospective study. Public Health Nutr..

[39] Keehan S.P., Cuckler G.A., Sisko A.M., Madison A.J., Smith S.D., Stone D.A., Poisal J.A., Wolfe C.J., Lizonitz J.M. (2015). National health expenditure projections, 2014–2024: Spending growth faster than recent trends. Health Aff. (Millwood).

